# Therapeutic potential of popular fermented dairy products and its benefits on human health

**DOI:** 10.3389/fnut.2024.1328620

**Published:** 2024-02-28

**Authors:** Gul Naz Saleem, Ruixia Gu, Hengxian Qu, Gul Bahar Khaskheli, Imran Rashid Rajput, Muhammad Qasim, Xia Chen

**Affiliations:** ^1^College of Food Science and Technology, Yangzhou University, Yangzhou, Jiangsu, China; ^2^Key Lab of Dairy Biotechnology and Safety Control, Yangzhou, Jiangsu, China; ^3^Department of Animals Products Technology, Sindh Agriculture University, Tandojam, Pakistan; ^4^Lasbela University of Agriculture, Water and Marine Sciences, Uthal, Pakistan; ^5^Microelement Research Center, College of Resources and Environment, Huazhong Agricultural University, Wuhan, Hubei, China

**Keywords:** therapeutic, dairy food, milk, fermented products, probiotics, koumiss, human health

## Abstract

In the current arena of time, the transformation of society has improved the standard of living in terms of lifestyle and their nutritional demands and requirements. The microorganisms under controlled conditions and the enzymatic transformation of dietary components are the processes that resulted in fermented foods and beverages. Fermented dairy products with high nutritional value are “the pearls of the dairy industry.” During fermentation, fermented dairy products produce bioactive compounds and metabolites derived from bacteria. Research indicates the beneficial effects of probiotics found in dairy products on human health is making lightning-fast headway these days. The utilization of lactic acid bacteria as probiotics for the prevention or treatment of disease has been a driving force behind the discovery of novel potential probiotics found in naturally fermented milk. Probiotics such as lactic acid bacteria and bifidobacteria found in fermented dairy products have a variety of health benefits, including innate immune enhancement, diarrhea treatment, inflammatory bowel disease, diabetes, Tuberculosis, and obesity, relieving irritable bowel disease symptoms, preventing cancer, improving lactose tolerance, lowering cholesterol, enhancing antioxidant activity, and antimicrobial activity against pathogens. This review aims to evaluate the therapeutic efficacy and nutritional and microbiological properties of popular fermented dairy products and their health benefits.

## 1 Introduction

Bioactive substances and vital nutrients abound in dairy products. The primary dairy product, milk, has lipids, sugar, and proteins including whey and casein (1). Yogurt is made by fermenting milk with certain bacteria and is renowned for its probiotics, which are good for the digestive system. Another derivative, cheese, varies in kind and aging procedure but delivers concentrated proteins, lipids, vitamins, and minerals (2). Fat-soluble vitamins and short-chain fatty acids are found in butter. The minerals calcium, phosphorus, and vitamins B12 and D included in these products are also vital for healthy bones. Dairy products also include bioactive peptides that have antioxidant and antibacterial qualities that may be beneficial to health (3). Moreover, dairy products such as milk can be fermented to produce yogurt and cheese etc. Fermentation is used for the conversion of carbohydrates into alcohol or acids via the action of microbes such as yeast and bacteria (4). This process improves the taste and shelf life of food as well as the availability of nutrients, adds beneficial microorganisms, facilitates digestion, and strengthens immunity (5). Yogurt, sauerkraut, kimchi, and kombucha are examples of fermented foods that have many health advantages such as they improve digestion, strengthening the immune system, improving gut health, and promoting nutrient absorption. They may also lower the risk of chronic diseases (2). Numerous nations utilize fermented milk products due to their health benefits. In emerging nations, particularly in Africa and Asia, most of their population uses fermented milk and products (6). Fermented foods can be described as food products or beverages produced by the controlled development of microorganisms and the enzymatic conversion of dietary constituents (7). The health advantages of fermented milk are contingent upon the functionality of living microbes, commonly known as starter cultures, in conjunction with the nutritional composition of the milk (8). These microbe’s presence enhances milk longevity by augmenting its acidity levels and facilitating the emergence of organoleptic characteristics, such as flavor and texture (9). Most of the microorganisms used in milk are probiotic and known as lactic acid bacteria (LAB) (6, 10). There is a possibility that certain LAB strains are employed as probiotics in the food industry. Lactic acid bacteria (LAB) play a vital role as dietary microorganisms, Lactic acid bacteria (LAB) are commonly obtained from diverse dietary sources, and strains exhibiting exceptional efficacy and strong competitive abilities are employed as probiotics. Recently, there has been a growing scholarly focus on extracting and analyzing lactic acid bacteria (LAB) from a diverse range of fermented food items and commodities.

**Figure 1:**
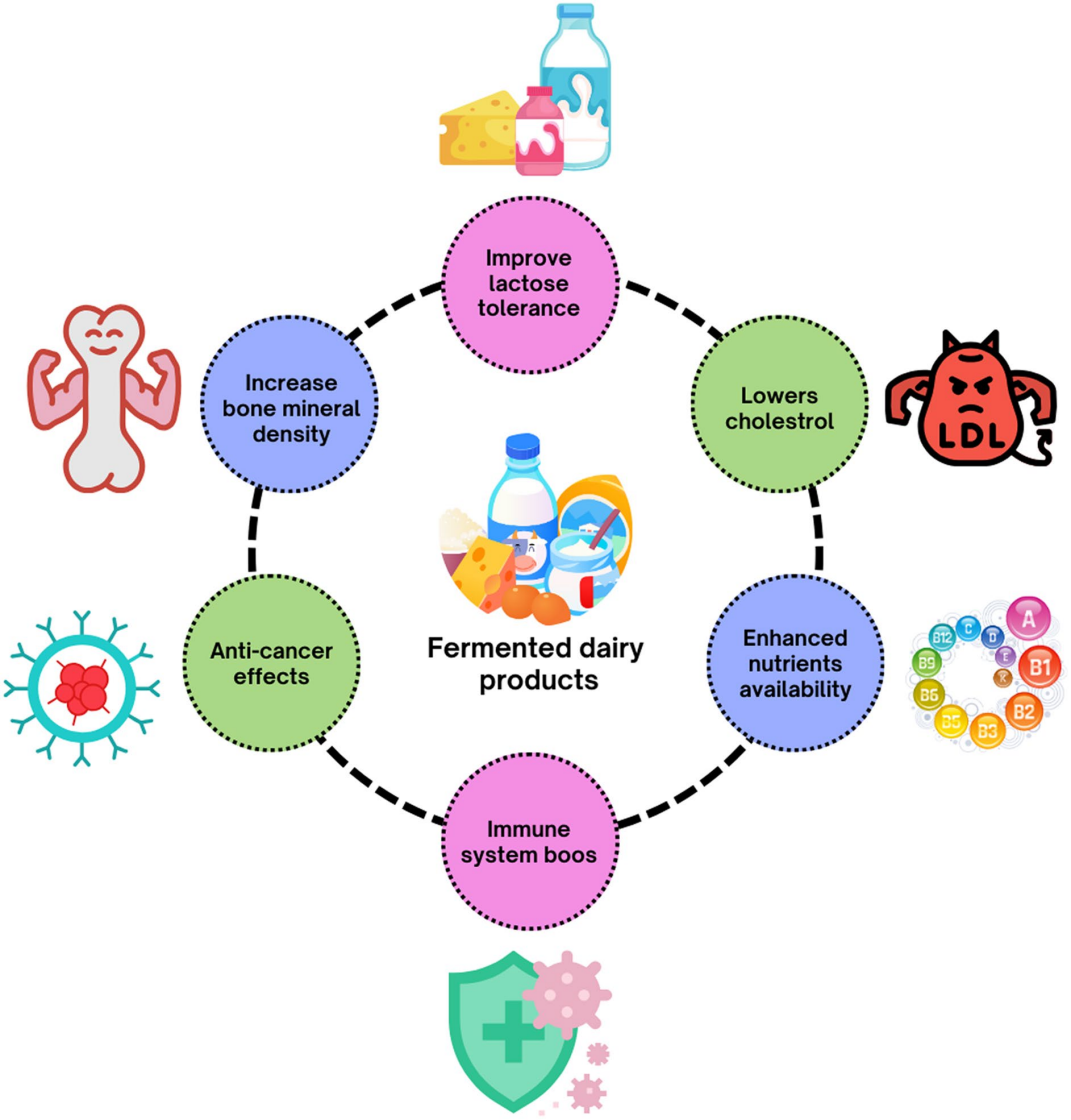
Therapeutic effects of fermented dairy products (probiotics).

The majority of microorganisms are “Generally Recognized as Safe (GRAS).” Globally consuming dairy products principally utilize the dietary sources for LAB to generate the milk into its unique and beneficial products (11, 12). In the past decade, the LAB has attracted the industry and frequently employed as probiotics, which offers health benefits to the host when taken in appropriate numbers (13). The utilization of LAB strains, namely *Enterococcus* spp., *Lactococcus* spp., and *Lactobacillus* spp., possessing antibacterial capabilities, has been employed in bio-control approaches aimed at diminishing mycotoxins and augmenting bioavailability (14, 15). Recent research has elucidated that probiotic lactic acid bacteria (LAB) strains had the potential to eliminate mycotoxins effectively. LAB has been shown to improve intestinal transit, keep intestinal flora in balance, and keep the acid–base balance in the colon. This helps to regulate the immune system and lower serum cholesterol levels. Improves the equilibrium of intestinal microorganisms to promote human health (16). Certain strains of lactic acid bacteria (LAB) have been observed to elevate the concentrations of pro-inflammatory markers such as TNF-α, IL-1β, and IL-6 while concurrently reducing the expression of anti-inflammatory markers (Arg 1, TGF-β, and CD206). This effect is achieved through the induction of macrophage polarization toward the M1 phenotype (17, 18). The worldwide interest in functional foods containing nutrients with potential health benefits in Europe, North America, and Asia accounts for up to 77% of the fermented milk and yoghurt business, which is presently worth €46 billion (19, 20). Among the fermented products, Yoghurt is also one of the world’s most widely consumed fermented dairy products owing to its health advantages beyond its essential nutritional value. Yoghurt is generally considered a nutrient-dense food due to its nutritional profile. A calcium-rich diet offers considerable quantities of calcium in the bioavailable form (21). Cheese is a globally recorded product made from the milk of ruminants via a mix of physical processes. Caseingniz and calcium are both essential for transforming milk into curd (22). In recent years, kefir’s significant health benefits have attracted the scientific community’s attention (23). In addition, Koumiss is a well-known dairy product made from fresh mare milk and contains a small amount of alcohol. It is naturally fermented with the original combination of yeasts and bacteria (lactic acid bacteria and yeast) (24). Mongolians use it to treat various diseases that have been globally well-known (25). Although numerous scientific studies have been reported on the therapeutic effects of dairy products on human health (Figure 1), this encompasses all the relevant data and mechanisms of dairy products on human health such as yoghurt, kefir, cheese, and koumiss due to their popularity and therapeutic effects. Also, this study seeks to describe and characterize popular fermented foods, their methods of action (including influence on microbiota), and their effects on human gastrointestinal health and illness.

## 2 Therapeutic effect of yogurt on human health

The origins of yoghurt may be traced back to the Middle East and Western Asia, where it has since become a staple diet for many people (26). Yogurt is a rich source of several critical minerals, including calcium and protein. Various studies documented the therapeutic effects of yoghurt (Table 1) and it is considered a healthy food because it is easy to digest and the body absorbs nutrients easily. According to the committee on the medical aspects of food policy, one cup of yoghurt (245 g) provides 40% of the reference nutrient intakes (RNI) for calcium, 40% for phosphorus, 10% for potassium, 10% for vitamin A, 30% for vitamin B2, and 60% for vitamin B12 for males and females aged 19–50 (COMA) (27). Yoghurt consumers have been demonstrated to have much lower rates of riboflavin, vitamin B-12, calcium, magnesium, and zinc deficits than non-yoghurt consumers (28). However, most studies on the health advantages of yoghurt focus on its live bacterial content (2). Yogurt is a carrier of probiotics and can be divided into two categories: regular culture yoghurt and bio- or probiotic yoghurt. Standard yoghurt is manufactured using *Lactobacillus delbrueckii* subsp. *bulgaricus* and *S. thermophilus* bacteria. Bio yoghurts are tic strains (29). Eating yoghurts may also promote microbial diversity in the intestine (28). It is also indicated for gastrointestinal problems such as inflammatory bowel disease and irritable bowel illness, immunological function, lactose intolerance, and produced by cultivating extra helpful microbes, mainly *Bifidobacterium* and *L. acidophilus* (2, 29).

**Table 1:** Health promising therapeutic effects of yoghurt consumption documented in human against some major pathological disorders.

Condition	Activity	Subject	Effect	References
Human model	BMD enhancement Osteoporosis Prevention	4,310 adults	Females with the highest yogurt intakes (>once per day serving) had higher BMD yogurt consumption decreased the risk of osteoporosis (i.e., 39% in female and 52% in males).	(43)
Human	Anticancer effect	116 males and 173 females	The administration of high-dose enriched yoghurt lowered colorectal cancer risk, with males showing a higher protective impact than females.	(62)
Human	Diarrhea prevention	Children aged 1–12 years	Probiotic and pasteurized yoghurts were tested for their efficacy in reducing antibiotic-associated diarrhea. Probiotic yoghurt has been reported to reduce AAD in children.	(37)
Human	Diabetes and liver disease prevention	40 patients	The experimental group of patients were given probiotic yoghurt (one cup per serving, three times per day for 14 days) had a significant effect on diabetes.	(58)
Human	lactose intolerance	55 patients	Yoghurt supplemented with *L. acidophilus* and *Bifidobacterium* sp. might safely and effectively reduced lactose intolerance.	(28)
Human clinical trial	Constipation	Pregnant women (treatment group and controlled group)	There was non-significant difference between the treatment and control groups, and both types of yoghurt improved digestive function.	(64)

### 2.1 Obesity

Obesity is an increased risk of chronic diseases and affects both industrialized and developing nations (30). According to the latest review and meta-analysis of 39 randomized controlled trials, probiotic fermented milk products might be used as adjuvant therapy to lower total cholesterol, LDL cholesterol, and triglycerides in the blood, particularly in men (31). Because of its probiotics, high protein content, low glycemic index, and calcium content, yogurt may help reduce weight. These elements support intestinal health, fullness, and effective metabolism, all of which lower caloric consumption frugis. Nevertheless, a person’s total diet and lifestyle determine how beneficial it is, thus going with basic, unsweetened versions is advised (32). Individuals who drank the most whole-fat yoghurt were substantially less likely to develop metabolic syndrome components such as abdominal obesity, hypertriglyceridemia, low HDL cholesterol, high blood pressure, and high fasting plasma glucose, according to Babio et al. (33). Yoghurt consumption was connected with a lower chance of gaining weight, according to a study that tracked three large cohorts of people for up to 20 years and included 120,877 obese men and women free of chronic conditions at the start of the trial. The authors postulated that shifts in the bacteria that live in the digestive tract could be responsible for observed variations in weight (34).

### 2.2 Diarrhea

It is a frequent, typically brief ailment that many people experience yearly. Ingestion of probiotics in the weeks preceding a trip has been shown to lower the incidence of traveler’s diarrhea by up to 15% (35). A meta-analysis of 63 randomized controlled studies indicates that probiotics may reduce the duration of diarrhea caused by bacterial, viral, or parasite diseases by around 25 h (36). Fox et al. (37) investigated the efficacy of a probiotic yoghurt (200 g/day) containing *Lacticaseibacillus rhamnosus* GG, *Bifidobacterium animalis* subsp. *lactis*, and *Lactobacillus acidophilus* in preventing antibiotic-associated diarrhea in children aged 1–12 with that of a pasteurized yoghurt. There is evidence that probiotic yoghurt decreases the prevalence of ADD in children. The effects of yoghurt for infants aged 6–24 months hospitalized with severe diarrhea were favorable. In addition to routine hospital treatment, at least 15 mL/kg/day of pasteurized cow milk yoghurt was administered orally to infants in the case group. According to the data, there were substantial improvements in the average number of hospitalized days, the frequency of diarrheal episodes, and weight gain (38). Probiotics, which restore gut flora balance, and lactic acid bacteria, which suppress pathogenic bacteria, are the main ways that yogurt helps manage diarrhea (39). While it is not a panacea for all diarrheas, it is simple to digest, helps with nutritional absorption, boosts immunity, and is appropriate for those with lactose sensitivity (40).

### 2.3 Osteoporosis

It is a serious disease caused by decreased bone mineral density (BMD) and is associated with a significantly increased fracture risk (41, 42). Evidence from the past suggests that consuming more calcium-rich dairy products may protect individuals against bone loss (21). Among the reviewed studies was an Irish study of over 4,000 individuals over the age of 60, in which a higher yoghurt intake was associated with a reduced risk of osteoporosis—a more significant effect than that observed with milk intake (43). Another researcher reported that fermented dairy products exhibit a notable impact on bone health in comparison to regular milk (44). In addition, probiotics have been found to impact the permeability of the intestinal wall. The gut microbiota plays a role in the breakdown of dietary minerals and can potentially enhance calcium absorption. The enhanced absorption of calcium may decrease the generation of parathyroid hormone, potentially causing a decline in bone reabsorption. The modulation of serotonin secretion may also increase bone growth (45). The major way that yogurt helps manage osteoporosis is because it contains a lot of calcium, which is good for strong bones. Enhanced with vitamin D, it also improves the absorption of calcium. Bone health is enhanced by the protein and other nutrients included in it. Yogurt is good, but it should only be used in conjunction with a whole osteoporosis treatment strategy that includes food, activity, and maybe medicine (46).

In the Framingham offspring study with 2,506 male and female participants, those who consumed a considerable amount of yoghurt (more than four servings per week) had a higher bone mineral density (BMD) at the trochanter than those who did not consume yoghurt (47). However, other locations in the skeleton showed no significant relationships. Additionally, research conducted on a cohort of 4,310 individuals found an association between the consumption of yoghurt and higher bone mineral density (BMD) and physical function ratings. Yoghurt was consumed by a significantly more significant proportion of females than men, and the average amount of yoghurt ingested by females daily was considerably more than that of males (0.42 vs. 0.32 servings per day, respectively). Consumption of yoghurt by females was an excellent predictor of bone mineral density across all areas, those females who consumed the most yoghurt (more than one serving per day) had higher total hip and femoral neck bone mineral density compared to those who consumed the least yoghurt (one serving per week or never). Men who did not consume yoghurt had a vertebral BMD that was 4.1% higher than those who consumed it but drank less of it. There was a correlation between increasing one’s consumption of yoghurt by one unit and a reduction of 31% in the incidence of osteopenia, 39% in the risk of osteoporosis in females, and 52% in the risk of osteoporosis in males (43).

### 2.4 Lactose intolerance

People with lactose intolerance have stomach problems when they drink milk or eat milk products because they do not have enough lactase activity in their small intestines to digest the milk sugar lactose (48). Many studies have shown that lactose-intolerant can benefit from consuming fermented milk products because some lactic acid bacterial strain products secrete lactase into the digestive system (49). Recently in-depth research found that probiotics such as *Lactobacillus* spp., *B. longum*, and *B. animalis* had beneficial impacts, justifying the usage of probiotic yoghurt comprising *L. acidophilus* and *Bifidobacterium* spp. (50). Another study found that probiotic yoghurt supplemented with *L. acidophilus* and *Bifidobacterium* spp. may safely and successfully lower lactose intolerance symptoms and HBT. As a result of this discovery, our probiotic can be suggested as the therapy of choice for lactose intolerance in patients (28). Low levels of the enzyme lactase induce lactose intolerance, which makes it difficult to digest lactose in dairy products. Because yogurt has less lactose, bacteria assist in digestion, and tolerance levels vary, it might be beneficial (51). For those with severe intolerances, lactose-free alternatives like Greek yogurt are especially good. Moreover, supplements might help with digestion (52).

### 2.5 Diabetes

In 2015, 8.8% of the world’s adults, or 415 million people, had diabetes. By 2040, this number is expected to rise 10.4%, or 642 million people (53). Dairy products are an excellent source of vitamins, magnesium, vitamin D, and certain fatty acids. Moderate dairy consumption is associated with a reduced incidence of type 2 diabetes, according to research (54). The relationship between yoghurt intake and health advantages is more stable than those of other dairy products, for which results have been inconsistent. Daily yoghurt consumption may also lessen the incidence of cardiovascular disease and type 2 diabetes (55, 56). Two meta-analyses of prospective cohort studies found that daily intake of yoghurt decreased the risk of developing type 2 diabetes by 18 and 14% (54, 57). It is considered that beneficial bacteria in yoghurt may decrease inflammation or enhance the body’s natural insulin effectiveness. In addition to reducing the intestinal flora imbalance that patients with chronic liver disease experience, probiotic yoghurt significantly impacts persons with chronic liver disease. Forty patients in the experimental group were given probiotic yoghurt containing *Bifidobacterium bifidum*, *Lactobacillus acidophilus*, *Lactobacillus delbrueckii* subsp. *bulgaricus*, and *Streptococcus thermophilus* three times daily for 14 days. This medication effectively decreased liver conditions, especially in diabetes (58). Because of its high nutritional content, low glycemic index, and probiotics for gut health, yogurt may help treat diabetes. It is a fantastic alternative to meals with a higher GI and may help lower inflammation. It’s preferable to choose low-sugar options, such as Greek yogurt. Nevertheless, it ought to support a well-balanced diet (59).

### 2.6 Cancer

The World Health Organization ranks colorectal cancer (CRC) as the third most frequent form of the disease overall and the 2nd largest cause of cancer-related death (60). Yoghurt has an anti-cancer impact on the mucosa of the colon and rectum due to its microbial components (61). The 10 male participants in the European Prospective Investigation into Cancer and Nutrition (EPIC) cohort in Italy reaped the benefits of the protective effect of high-dose supplemented yoghurt to a greater extent than the nine female individuals (62). According to the findings of Margolis et al. (56), post-menopausal women who drink a greater quantity of yoghurt had a lower risk of acquiring diabetes. In a past study that included 1,183 men and women in Australia between the ages of 39 and 65, a cross-sectional examination revealed a significant connection between the use of low-fat yoghurt by males and self-reported measures of memory recall and social functioning (63), but it is still not fully understood that how the yoghurt acts as anti-cancerous effects.

### 2.7 Gut health and respiratory infection

A clinical investigation observed whether probiotic yoghurt may alleviate some pregnant women’s constipation problems. A randomization procedure and varied controls were utilized in this study. The experimental group drank 300 grams of *Bifidobacterium* and *Lactobacillus*-containing yoghurt, while the control group ate regular yoghurt containing *Lactobacillus delbrueckii* subsp. *bulgaricus* and *Streptococcus thermophilus*; each group carried out the experimental procedures in equal time, and neither group experienced any adverse effects. Regarding the enhancement of bowel function brought about by probiotics and plain yoghurt, there was no discernible difference between the treatment and control groups (64). Makino et al. (65) showed that regular ingestion of yoghurt containing live culture might increase the adult ‘s resistance to respiratory infections, especially in a cold atmosphere. Kefir is a little sour and alcoholic fermented milk product with a creamy consistency (66). In the experimental group, 40 individuals consumed probiotic yoghurt three times daily for 14 days. It contained *Streptococcus thermophilus*, *Bifidobacterium bifidum*, *Lactobacillus acidophilus*, and *Lactobacillus delbrueckii* subsp. *bulgaricus*. This medication considerably improved hepatic conditions, especially diabetes (67).

As yogurt is rich in probiotics, it enhances the gut health and indirectly boosting the immune system. This phenomenon aids in controlling respiratory infections, highlighting the gut-lung axis interplay Daniel. The probiotics load in the yogurt can also directly inhibit the pathogens and reduce inflammation, thereby contributing to overall human health and respiratory infection resistance (68).

## 3 Therapeutic effect of kefir on human health

Kefir has a carbohydrate content of 3%, a lipid content of 3.5%, a protein content of 3%, and an ash content of 0.7%. When it is safe to consume, kefir has a wealth of vitamins (69). During fermentation, acid coagulation and proteolysis improve protein digestion. The amino acid profile of kefir is identical to the amino acid profile of fermented milk (70). Kefir has elevated ammonia, serine, lysine, alanine, and threonine (71), as well as tryptophan, valine, lysine, methionine, phenylalanine, and isoleucine (72). Kefir is a rich source of magnesium, calcium, and phosphorus. Milk kefir also contains essential minerals such as Zinc, Copper, Manganese, Iron, Cobalt, and Molybdenum (73). Kefir, and kefir-related strains, have been shown to have a significant impact on health as presented in Table 2.

**Table 2:** Invitro and *In vivo* evidences of therapeutics benefits offered by kefir “a functional dairy foods.”

Condition	Activity	Subject	Effect	References
*In vivo*	Anticancer activity	Mice	The consumption of milk kefir and soymilk kefir has been shown to considerably suppress the formation of tumors in mice and dramatically boost IgA levels.	(86)
Vitro	Antioxidant activity	–	Kefir has a strong affinity for binding the superoxide radical and the 1,1-diphenyl-2-picrylhydrazyl (DPPH) radical, in addition to its ability to suppress linoleic acid peroxidation.	(93)
*In vivo*	Gut health improvement	Mice(two groups of 6)	Bifidogenic growth is one of the probiotic effects of kefir on the gut’s bacterial population.	(77)
Vivo	Antimicrobial properties	Mouse	Antagonistic activity was shown to protect against potentially dangerous microbes (*Salmonella typhimurium*).	(80)
*In vitro*	Antimicrobial properties	–	Researchers have discovered that *Lentilactobacillus kefiri* B6 is immune to the harmful effects of bile and exhibits antipathogenic capabilities.	(95)
Vivo	Inflammatory bowel disease (IBD)	Rat	Reduces the amount of diarrhea and damage to the mucosal wall caused by the illness at the macroscopic level.	(96)

### 3.1 Gut health

Kefir provides beneficial bacteria with probiotic effects. Several bacterial species isolated from kefir exhibit extraordinary resistance to the gastrointestinal system’s low pH and bile salts, as well as the capacity to adhere to intestinal mucus (74). Furthermore, the bacteria in kefir may produce antimicrobial substances such as organic acids and bacteriocins (75) and interfere with the adhesion of pathogenic bacteria in the intestinal mucosa, possibly contributing to gut health improvement (76). In the intervention group (*n* = 6), it was similarly found *in vivo* with a daily dose of 0.75–1 mg of dairy. The animal units were divided into two groups of six, Kefir a probiotic affects gut bacterial populations by boosting bifidogenic bacteria (77). Additionally, kefir regulates digestion, strengthens the immune system, and has anti-inflammatory properties. It helps in lactose digestion, rehabilitates the gut lining for better nutrient absorption, and reduces allergy and asthma risks, making it beneficial for overall gastrointestinal wellbeing (78).

### 3.2 Antimicrobial properties of kefir

According to research conducted in the early twentieth century, the good impact of regular yogurt eating, including lactic acid-producing microorganisms, on life expectancy was related to the struggle between LAB and dangerous pathogens. Kefir is said to be bactericidal in Gram-negative bacteria, although it is more effective against Gram-positive bacteria (79). A mouse model of *Salmonella typhimurium* infection control was used in research conducted by Cordeiro et al. (80). The results of that investigation have shown that fermented dairy beverages that are widely eaten protec against pathogenic bacteria. Oral kefir feeding to mice for 28 days resulted in increased levels of *Lactobacillus* and *Bifidobacterium* in the animal feces while simultaneously reducing the amount of *Clostridium perfringens* was seen (81). Studies have shown that *Shigella*, *Staphylococcus*, *Helicobacter pylori*, *Escherichia coli*, *Enterobacter aerogenes*, *Proteus vulgaris*, *Bacillus subtilis*, and *Micrococcus luteus* all exhibit traits that are hostile to one another (79, 82, 83). Kefir was also shown to be antibacterial when tested against *Candida albicans*, *Escherichia coli*, *Staphylococcus aureus*, *Salmonella typhi*, and *Shigella sonnei* (75). It exhibits antimicrobial properties due to being loaded with probiotics, organic acids, and bioactive compounds which work together to inhibit harmful bacteria, fungi, and viruses, lower pH, and outcompete pathogens for nutrients and boost the immune system (84).

### 3.3 Cancer

**Figure 2:**
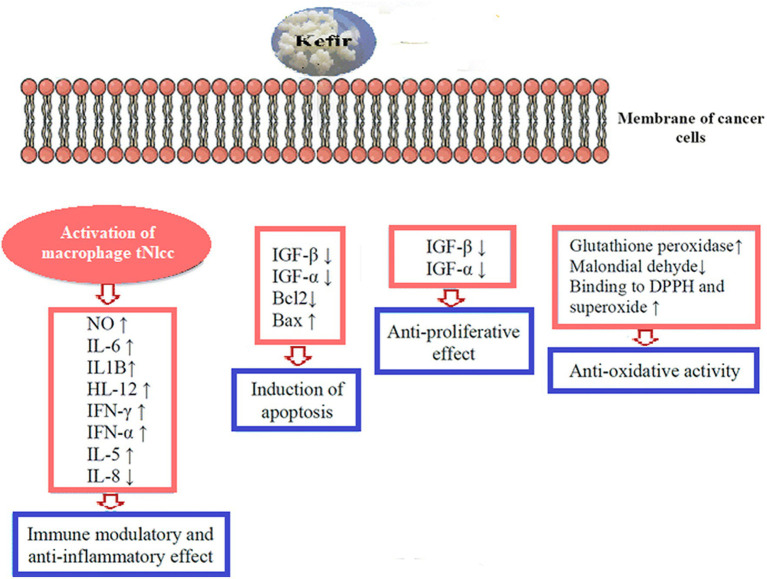
Mechanism of action of Kefir for anticancer activity.

The second leading cause of mortality worldwide is cancer. However, Weir et al. (85) reported that a healthy diet might prevent up to 50% of cancers. Therefore, kefir’s probiotics are vital as a possible co adjuvant treatment or cancer prevention. Throughout the years, several *in vitro* and *in vivo* studies have demonstrated the anti-cancer potential of kefir. The mechanisms by which kefir exerts its anticancer effect is shown in Figure 2. Numerous cancer forms, including hematological malignancies, breast cancer, digestive tract tumors, and sarcoma, were examined for the anti-carcinogenic efficacy of kefir and kefir fractions. In 2002, Liu et al. reported that milk kefir and soymilk kefir intravenously to mice with sarcoma. After 30 days of ingestion, both forms of kefir effectively inhibited tumor development by inducing apoptotic cell death in tumor cells. They considerably boosted IgA levels in mice, suggesting that both forms of kefir possess anti-cancer capabilities and enhanced mucosal resistance to gastrointestinal infection (86). Kefir supernatant has been studied as a potential adjuvant for doxorubicin (DOX) therapy because of its chemo-sensitizing effects on multidrug-resistant (MDR) human colorectal cancer cells (HT-29) (87). Kefir and DOX contributed to an increase in intracellular ROS buildup in HT-29 MDR-developed cells, resulting in a down regulation of ABC transporters. Researchers observed that all of the bacterial strains that were isolated from kefir have a high potential to adhere to mutagens (>985%), which may then be eliminated via feces, therefore maintaining colonocytes (88). In addition, Khoury et al. (89) found that kefir induces apoptosis and inhibits the development of HT 29 and Caco 2 colorectal cancer cells. Kefir also suppresses the growth of colorectal cancer cells.

### 3.4 Antioxidant properties

Several biochemical experiments have been used to determine that kefir has antioxidant capabilities (90, 91). These qualities changed during the fermentation and aging processes. Kefir exhibited more potent antioxidant effects than vitamin E when tested in a mouse toxicity study with carbon tetrachloride (CCl_4_) conducted by Güven et al. (92). Liu and colleagues investigated the antioxidant capacity of kefir produced from goat and cow milk. Many researchers found that kefir has the potential to bind DPPH and superoxide radicals as well as reduce the amount of linoleic acid that was peroxidized (93). Additionally, the DPPH radical scavenging ability and the inhibitory effects on linoleic acid autoxidation were improved. Consequently, the TPC and its ability to inhibit ascorbate autoxidation were found to be better upon kefir utilization (94).

## 4 Health benefits of koumiss

Koumiss has long been considered a great meal and beverage with potent medicinal properties (97). Koumiss was traditionally being produced by inoculating raw, unpasteurized mare’s milk in tanks (98). The important microorganisms in Koumiss are lactic acid bacteria, which convert lactose into lactic acid, and yeast, contributing to the 3–8-h fermentation (24). Koumiss therapeutic services are typically supplied by small and medium-sized lodging businesses, especially in rural parts of Asian nations (99). Koumiss has many fatty and protein-containing nutrients, vitamins, amino acids, carbohydrates, and trace mineral elements (100). Due to its lower fat content, mare milk has lower calories (480 kcal kg/l) than human or bovine milk. Furthermore, it is abundant in vitamins C, A, E, D, B1, B2, and B12, trace minerals, and antibiotics (101, 102). Koumiss being a well-known probiotic beverage, contains all of the essential amino acids required by humans, including proline, lysine, tyrosine, valine, and leucine (102). Koumiss’s microflora consists primarily of LAB cultures, including *Lactobacillus delbrueckii* subsp. *bulgaricus, Lactiplantibacillus plantarum*, and *Lactobacillus helveticus L. casei L. acidophilus* and two species of yeast, *Kluyveromyces marxianus* and *Saccharomyces cerevisiae* (103). Koumiss’s nutritional and therapeutic characteristics qualify it as a functional food (104). Health promoting benefits of Koumiss are highlighted in Table 3, out of those some of them are due to its high probiotic content, antibacterial and antifungal properties, regulation of immunity, maintenance of a healthy gastrointestinal tract, regulation of cholesterol and blood sugar levels, regulation of blood pressure, and induction of several essential vitamins etc. As a functional food component, Koumiss has generated considerable industry interest due to its great potential in treating several health disorders (105). Recently, the intake of koumiss in meals has been regarded as a healthy eating trend in European countries (106).

**Table 3:** Health promising therapeutic effects of Koumiss documented against some major pathological disorders.

Condition	Disorder	Effect	References
Vivo (Mice)	Toxoplasmosis infection	Chronic infection models demonstrated that Koumiss dramatically decreased the frequency of brain cysts and impact the resistance of the gut microbiota to *T. gondii* infection	(109)
Rats	Immune system disorders	Mare milk or mare milk koumiss enhances normal immunological activities, regulates cell immune capacities	(120)
*In vivo* (T.B patients)	Tuberculosis	It is documented that patients treated with Koumiss for T.B has 60–91% recovery rate	(118)
*In vivo* (Patients)	Hyperlipidemia	At 30th and 60th day, relative quantities of 10 important differential metabolites increased significantly in patients which linked to lipid reducing potential	(104)
Human	Oxidative stress	Kumiss supplement reduced plasma 8-OH-2-deoxyguanosine, glutathione levels, tissue oxidative stress index and increased antioxidant activity of tissues	(121)

### 4.1 Antibacterial spectrum

The inhibitory impact of lactic acid antibodies on pathogenic microbes may be clearly shown when tested *in vitro*. Yeasts are the most prevalent microorganisms in Koumiss; they are essential for the fermentation process and are responsible for offering therapeutic advantages to the consumers. According to research carried out by Etienne-Mesmin et al. (107), certain strains of yeast can inhibit the growth of *E. coli* by producing antibacterial compounds, such as killer toxins and organic acids, during their metabolic processes. It has been determined that yeast strains and Koumiss have antibacterial substances that are efficient against *E. coli*. Four antibacterial substances have been identified from Koumiss yeasts (108). Three Gram-negative bacteria, three Gram-positive bacteria, and five pathogenic *Escherichia coli* strains were used to test the antibacterial properties of beneficial yeast (*Kluyveromyces marxianus* and *Saccharomyces cerevisiae*). The antibacterial chemicals produced by yeasts in Koumiss are found to be more effective against Gram-positive than Gram-negative bacteria. Koumiss considerably affected chronic *T. gondii* infection in mice and might ameliorate acute *T. gondii* infection signs. Surprisingly, chronic infection models demonstrated that Koumiss dramatically decreased the frequency of brain cysts in mice (*p* < 0.05), improved amyloid deposition in the hippocampus (*p* < 0.01), and decreased the levels of IFN- and TNF (*p* < 0.01, *p* < 0.05), also, Koumiss may impact the resistance of the gut microbiota to *T. gondii* infection. The research provides additional evidence for the development of safe and effective anti-*T. Gondii* strategies and expands our understanding of the potential use of Koumiss (109).

### 4.2 Hyperlipidemia

The World Health Organization (WHO) predicts that by 2030, heart disease will continue to be the leading cause of death, affecting around 23.6 million people. Hypercholesterolemia is one of the leading hazards of CHD (110). Hyperlipidemia is a condition of lipid metabolism characterized by high total cholesterol and triglycerides in the blood (TGs). The pharmaceutical industry has developed several treatments for these disorders. However, long-term use of hyplipidemic drugs is associated with several deleterious consequences (111).

Consequently, dietary modification has become an intriguing alternative treatment method for efficiently reducing blood lipids (112). Koumiss is excellent to consume due to its abundant minerals and probiotic microorganisms (113). Moreover, Dönmez et al. (102) found that drinking Koumiss decreased the levels of TGs and cholesterol in the blood of individuals with hyperlipidemia. Zhang et al. (114) also discovered that the probiotic strain derived from Koumiss could reduce dyslipidemia and hyperlipidemia. Another research (13 patients aged 43–57 treated with kumiss) revealed that frequent ingestion of koumiss regulated not only the blood cholesterol level but also the makeup of the gut flora in individuals with hyperlipidemia (104). Before and after koumiss medication, the fecal metabolomes of hyperlipidemia patients who ingested Koumiss daily were examined. At days 30 and 60, relative quantities of 10 important differential metabolites (ursolic acid, linoleic acid, stearic acid, −tocotrienol, −tocotrienol, alanine, tyrosine, sphingosine, acetate, and butyrate) increased significantly. These results showed that the 10 discovered metabolites were probably linked to the observed lipid-lowering effect (115).

### 4.3 The treatment of tuberculosis

Tuberculosis (TB) is one of the most prevalent infectious diseases globally, with 1, 2 million fatalities expected in 2019 (116). A Russian physician identified the efficacy of sour mare’s milk in treating 41 patients with pulmonary TB, observed remarkable patient improvement, and decided to include Koumiss in medical practices (117). Initially, Mongolian physicians used Koumiss to cure TB and included it in their therapeutic practices. At the Ximeng Mongolian Medical Research Institute, Koumiss effectively treats TB every summer and fall. In actual practice, treatment with Koumiss for TB patients has resulted in a 60–91% recovery rate, as validated by X-rays and tuberculosis tests. The lack of symptoms is indicative of therapeutic effectiveness (118). To avoid TB, the Cossacks have incorporated Koumiss into military meals (119).

### 4.4 Antioxidant potential and immune system boosting

**Figure 3:**
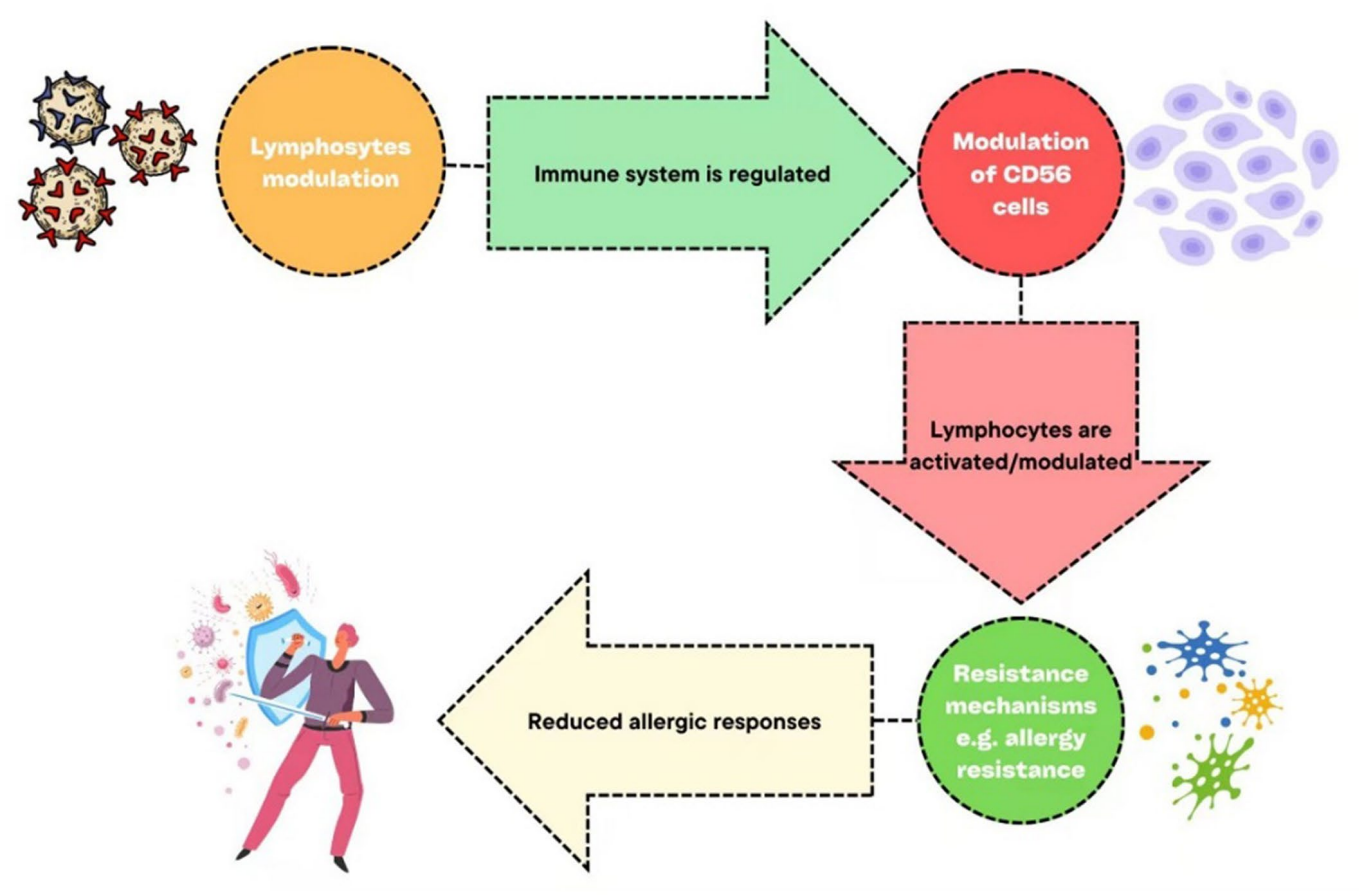
Role of koumiss in immune system enhancement.

About 80% of the body tissues are located in the intestines, and a regular meal of Koumiss boosts the immune system. Bacteria from fermented meals create substances that penetrate the intestinal wall and promote the immune system’s production of immune cells (25). Mare milk improves the weight of immune organs in rats, enhances normal immunological activities, regulates cell immune capacities, and regulates abnormal immune systems in bodily fluids (120) (Figure 3). The immunomodulatory and anti-inflammatory properties of kefir are very important and will be effective through the following mechanisms.

Many illnesses are connected to oxidative stress. It was discovered that Dimethylhydrazine (DMH) induced oxidative stress increases plasma 8-OH-2-deoxyguanosine, tissue oxidative stress index, and total oxidant capacity. It was discovered that the Kumiss supplement reduced these levels, increased the overall antioxidant capacity of the tissue, and decreased glutathione levels (121). The effect of lactobacilli on the suppression of lipid peroxidation was investigated. As various regions of the LAB are rooted in the duodenum, which is essential for releasing intracellular contents, the inhibitory effects of cell free intracellular extracts on lipid peroxidation have also been investigated. In all investigated strains, weak cells and internal CFE display antioxidative activity. However, intracellular CFE suppressed linoleic acid peroxidation at a considerably higher rate than weak lactobacilli cells recovered from Koumiss samples (122).

## 5 Health promoting effects of cheese

In conventional cheese production, a coagulating agent such as rennet, acid, heat and acid, or a combination thereof is utilized to transform aqueous milk into a semisolid state (123). Although cow’s milk is the typical ingredient in the production of cheese, sheep or goat milk is also utilized in the production of some varieties (124). Other agents may be incorporated alongside the starter, contingent upon the specific cheese variety and manufacturing conditions (125). In the context of maturation and storage, it is not necessary to mature fresh cheeses, including cream and cottage cheese, but it is necessary to mature hard cheeses, including Swiss and cheddar. In general, aged cheeses are produced using rennet curd. Unique flavors develop in a fresh clot during maturation due to the action of probiotics and enzymes (126).

### 5.1 Effect of rennet from different source on cheese

The wide variety of coagulants that can be employed to coagulate milk into a gel structure is referred to as rennet. Bovine calf rennet, predominantly composed of chymosin, has conventionally been employed in the production of cheese (127). However, the expansion of the cheese industry and the constrained availability of calf rennet have prompted an exploration of alternative calf rennet. An additional source of rennet has been identified (128). Table 4 summarizes the different sources of rennet used for cheese production. The introduction of the bovine chymosin coding sequence into microorganisms has facilitated the widespread availability of fermentation-produced chymosin (FPC) (129). A number of researchers have been exploring rennet substitutes from plants and some of which have been applied to industrial-scale cheese production with control conditions (130, 131).

**Table 4:** Sources of milk-clotting enzymes used in cheese making.

Source	Rennet enzyme	References
Animal	Porcine pepsin, bovine pepsin, and chicken pepsin	(132)
Microbial	*Mucor miehei*, *Mucorpusillus*, *Cryphonectria parasitica*, *Endothia parasitica*, and *Rizomucor miehei*	(128, 133)
Plant	*Cynara cardunculus*, *Carica papaya*, *Ananas comosus*, and *Albizia julibrisiin*	(134–136)

The use of rennet can impact the first breakdown of proteins. In a study conducted by Sheehan et al. (133), it was demonstrated that Mozzarella cheese with lower fat content, produced using *R. pusillus* proteinase, exhibited elevated amounts of pH 4.6 soluble nitrogen during the ripening process. This was in contrast to cheeses manufactured with either FPC or *R. miehei* proteinase. Prior studies (137, 138) have already documented the influence of FCC on the characteristics of Cheddar and Mozzarella. The C/P ratio of FCC is significantly higher than that of bovine chymosin, indicating a more excellent milk clotting action and overall proteolytic activity. According to Bansal et al. (139), after 150 days of ripening, FCC produces full-fat Cheddar cheese that is firmer and requires more chewing. This cheese has a lower level of initial breakdown of proteins and has reduced bitter and brothy flavors. Cheddar cheeses that are low in fat and created with FCC exhibit comparable characteristics of being firmer, more resistant to chewing, and having a reduced bitterness compared to cheeses made with FPC, as stated by Govindasamy-Lucey et al. (140). The cheeses manufactured with reduced rennet concentration exhibited a slower rate of increase in the concentration of 12% trichloroacetic acid soluble nitrogen during ripening. There was no significant impact on the concentration of αs-CN and the hardness or springiness of the cheese. A study conducted by Moynihan et al. (137) discovered that low-moisture part-skim Mozzarella cheese exhibited comparable hardness despite variations in rennet concentrations during its production. Plant rennets have become a subject of growing interest in cheese industry, due to their easy availability and simple purification processes. Plant based rennet sometime effects sensory attributes of final products but the selection of appropriate milk or its ultrafiltration, the mixture of coagulants as well as the increase of salting time of cheese during ripening could be efficient ways to improve texture and reduce bitterness (141).

### 5.2 Therapeutic effect of cheese on human health

Cheese is a globally popular dairy food product, particularly among young peoples. Historically, cheese has largely concentrated milk protein with the extended shelf life. It is very essential to the healthy American, Asian, and European diet (142). Numerous health benefits of cheese have been mentioned in Table 5. It is a very desirable food product due to its great nutritional content, the flexibility of its applications, and the organoleptic qualities (143). Cheese is a source of vital nutrients, including proteins, fats, minerals, and vitamins (144). The high fat cheese and protein content make it an energy-dense, nutrient-rich diet ideal for our laborious ancestors (145). It is widely proven that cheese supplies almost all necessary amino acids above the amounts recommended for infants or adults (146). Lactoferrin is the multifunctional glycoprotein that ranges from 672 μg g^−1^ (soft cheese) to 1,218 μg g^−1^ (semi-hard cheese) in the cheese (147). These proteins have several physiological functions, such as host defense against diverse range of pathogens, iron homeostasis, anticancer, antiviral, and anti-inflammatory activities (148). It is rich in fat-soluble vitamins such as vitamin K2 (149, 150), vitamin A, and vitamin E (151). Cheese is neutral food category that may be included in a balanced diet (152). Calcium intake from it, not only aids in maintaining strong bones but also significantly lowers blood pressure and aids in weight loss when combined with low-calorie meals (145).

**Table 5:** Therapeutic effects of cheese in major health disorders.

Condition	Disorder	Effect	References
Clinical trial (40 patents)	Inflammatory autoimmune condition (RA)	Consumption of 30 g/day of probiotic cheese each day for 12 weeks reduces inflammation and improves the gut flora, resulting in beneficial effects on the severity and symptoms of rheumatoid arthritis	(180)
*Vitro*	Oxidative stress	It has been demonstrated that the WSPs extract from Cheddar cheese effectively scavenges free radicals and inhibits radical-mediated oxidation in Caco-2 cells	(191)
Human (two groups of children)	Dental caries	In the context of diet counseling for children, cheese might be recommended as a preventive measure due to its ability to avoid the fall in salivary pH caused by sugar consumption.	(194)
Human	Age related deteriotion of the immune system	The consumption of cheese containing *L. rhamnosus* HN001 and *L. acidophilus* has been demonstrated to enhance the immune response of healthy old age persons	(181)
	Cardiovascular disease (CVD)	Cheese contains saturated fatty acids associated with elevated LDL cholesterol levels. LDL cholesterol is considered an indicator of cardiovascular disease risk (CVD)	(164, 195)

### 5.3 Lactose intolerance

Ripened cheese is lactose-free, rendering it is appropriate for the dietary needs of individuals who are lactose intolerant. A portion of the lactose is initially removed with the whey during the cheese maturation process; the remaining lactose is fermented into lactic acid, acetic acid, diacetyl, acetaldehyde, ethanol, and CO_2_ (153). The absence of lactose in matured cheese is a benefit for the majority of adults (154). Around 70% of adults worldwide suffer from lactose intolerance in maturity; milk consumption induces a variety of unpleasant symptoms, including abdominal pain, diarrhea, nausea, and flatulence (155). It is not, however, essential that these individuals abstain from dairy products. With the rare exception of soft cheese and fresh cheese, all other varieties of cheese do not contain lactose. Consequently, individuals with lactose intolerance are able to partake in the consumption of these cheeses, which support a nutritious diet by virtue of their essential components, including calcium (156).

### 5.4 Diabetes

The impact of the maturation process on the enhancement of the advantageous properties of cheese has been the subject of research utilizing animal models. One study administered diabetic db/db C57BL/J mice to various varieties of cheese that had ripened for 35 days; the effects were assessed using blood profiles, hepatic lipid content, and glucose tolerance (*p* < 0.05). Consumption of 35-day-ripened cheese enhanced glucose tolerance significantly without affecting insulin secretion, resulting in a substantial reduction in lipid peroxide markers (mRNA expression of TBARS and NADPH-oxidase) in fatty tissues, with no discernible impact on body weight, food intake, or fat mass. Furthermore, a substantial reduction was observed in the hepatic lipid content of rodents (157).

### 5.5 Hypo salivation and dental caries

Cheese containing probiotic bacteria provides several health benefits, such as; improved oral hygiene due to less hypo salivation and dry mouth (158). It has been shown that acids produced by plaque bacteria during the fermentation of carbohydrates and starches because dental caries by dissolving tooth enamel. Caries is still the most prevalent dental disease (159), despite improving its incidence due to better prevention. Chewing a piece of cheese after consuming a sugary food quickly returns plaque pH to neutral (160) Cheese helps in management of hypo-salivation and dental caries through the stimulation of saliva production, neutralizing the oral pH, and providing calcium and phosphate minerals for tooth enamel remineralization. Its antibacterial properties stimulate anti cariogenic bacteria, while proteins reduce enamel demineralization through the formation of protective layer against acid and further dental decay (161, 162). According to the findings of Jensen et al. (163) not all cheeses are equally effective at preventing a fall in the pH of plaque. The preventive effects of fresh and young cheese appear lower than those of aged cheese.

### 5.6 Hypertension and cardiovascular disease

However, cheese also includes a significant amount of saturated fatty acids (SFAs). Blood LDL-cholesterol levels are typically viewed as an indication of the risk of cardiovascular disease. SFAs have been related to elevate LDL-cholesterol levels (CVD) (164). Cheese has been linked to an elevated risk of cardiovascular disease due to the presence of saturated fatty acids in its composition. Regular consumption of whole milk, sour milk, or cheese (≥ 1 serving•d − 1) was found to be negatively correlated with weight gain and to have a reduced risk of cardiovascular disease (CVD) (165). Particularly pertinent in this circumstance appear to be calcium and bioactive peptides. Allender et al. conducted a meta-analysis of 28 studies and found that calcium supplementation significantly decreased systolic blood pressure in both hypertensive and non-hypertensive participants. Cheese contains an abundance of bioactive peptides. One of the most intriguing and extensively researched biological functions among these peptides is their ability to inhibit angiotensin-converting enzyme (ACE). By inactivating the hypotensive effect of bradykinin and facilitating the conversion of angiotensin I to the highly potent vasoconstrictor angiotensin II, ACE is a crucial enzyme in the regulation of blood pressure. Peptides that inhibit the activity of ACE have been found to exert a beneficial impact on hypertension (166). Numerous studies have demonstrated the ACE-inhibitory properties of diverse cheese variants, attributing this effect to a variety of bioactive peptides (167, 168). Within this collection of ACE-inhibiting peptides, the tripeptides valyl-prolyl-proline (VPP) and isoleucyl-prolyl-proline (IPP) are among the most efficacious. The intestine readily assimilates peptides containing a C-terminal Pro-Pro sequence, and research has demonstrated that such peptides are relatively resistant to additional degradation by digestive proteases and peptidases (169). IPP and VPP are encrypted within the milk protein β-casein. Proteinases derived from *L. helveticus*, a lactic acid bacteria, possess the capability to liberate the two peptides above from fermented milk. Rats with spontaneous hypertension that were fed sour milk fermented with specific strains of *L. helveticus* exhibited a hypotensive effect in numerous *in vivo* studies (170, 171), in addition to humans (172–174). On the basis of the prevalence of *L. helveticus* as a strain utilized in cheese production and the intensive proteolysis that occurs during ripening, it was hypothesized that cheese might also exhibit ACE-inhibiting activity via the formation of VPP and IPP. The concentrations of VPP and IPP in 44 traditional cheese varieties (Swiss and non-Swiss cheeses) were found to vary considerably, ranging from 0 to 224 mg/kg^−1^ and 0 to 95 mg/kg^−1^, respectively. Low concentrations were observed in soft cheese, followed by average concentrations in semi-hard and hard cheeses, and finally, high concentrations in extra-hard cheeses (175).

### 5.7 Rheumatoid arthritis

Rheumatoid arthritis (RA) is the inflammatory auto-immune disease that, when prolonged, can lead to joint abnormalities, cartilage, and bone damages (176). It targets comparatively more women than men (177). It has been observed that the disruption of gut microbiota may alter immune function by increasing pro-inflammatory substances, which may exacerbate the symptoms of rheumatoid arthritis (178, 179). Several research articles in the past decades have shown that rheumatoid patients have changed microflora in their digestive systems (RA). It has been established that probiotic-based treatments dramatically enhance patients’ ability to recover. In this context, patients with rheumatoid arthritis participated in a clinical trial to examine the effects of probiotic cheese on inflammatory and anti-inflammatory markers, disease severity, and symptoms. It has been demonstrated that consuming probiotic cheese can reduce inflammation and enhance the microbiota in the stomach, both of which can have a beneficial effect on the severity and symptoms of the disease (180). Also, it has been established that healthy elderly adults who consumed cheese containing both *L. rhamnosus* HN001 and *L. acidophilus* had enhanced their immunological response. Furthermore, consuming cheese can also mitigate some of the immune system declined associated with aging. By producing specific metabolites, cheese containing specific LAB species can also inhibit the growth of certain toxigenic microorganisms. Some cheese LAB species exhibit antimutagenic and antigenotoxic properties also (181). Thus, it may decrease the risk of cancer (182). Cheese act as anti-arthritis agent via acting for enhanced calcium and protein absorption and support bone health and tissue repair (183).

### 5.8 Antioxidant activities

Several chronic diseases have been associated with oxidative stress (184). Food items have been fortified with synthetic antioxidants to prevent oxidative degradation, although they may represent possible health hazards (185). From this perspective, the present market needs more natural antioxidants with chemotherapeutic and preservation capabilities. The bioactive peptides extracted from fermented dairy products might be included in functional diets to lessen the risk of oxidative stress-related chronic illnesses (186). The antioxidant activity of bioactive peptides identified in Cheddar has been documented in multiple studies (187–190). In the study, assessed the antioxidant capacity of WSE in Cheddar cheese produced at various phases of ripening, both with and without adjunct cultures. The findings indicated that the antioxidant activity exhibited a dependence on the stage of maturation. The radical scavenging activity of 2,2′-casino-bis (3-ethylbenzothiazoline-6-sulfonic acid) (ABTS) was found to be higher in Cheddar cheese produced with adjunct cultures. The activity peaked in the fourth month of ripening (16.61 and 9.76 μmol of TE/mg of protein, respectively, for Cheddar cheese produced with and without adjunct cultures). The Trolox-equivalent antioxidant capacity (TEAC) exhibited a consistent upward trend throughout the ripening process and reached its peak value of 9.81 μmol of TE/mg of protein (187). According to an *in vitro* study by Huma et al. (191), the WSPs (water-soluble peptides) extracted from Cheddar cheese can protect the intestinal epithelium from oxidative stress due to its antioxidant properties. Caco-2 cells were shown to be resistant to radical-mediated oxidation when exposed to a WSPs extract produced from Cheddar cheese. In addition, cheese has a low lactose content, making it an excellent choice for lactose-intolerant individuals (192). The inseparable tradition and enjoyment of cheese should not be forgotten amidst this scientific research. Cheese act as antioxidant agent effects through its vitamins (A, E, K), selenium, and minerals provision abilities (193).

Further research is required to identify the primary determinants of the ability to produce cheese with consistently high concentrations of the two bioactive peptides described Cheese consumption was found to have a causally inverse relationship with cardiovascular diseases, including type 2 diabetes, heart problems, coronary heart disease, hypertension, and ischemic stroke, as well as cardiovascular biomarkers, such as; body mass index (BMI), waist circumference (WC), triglycerides (TG), and fasting glucose (FG). Additionally, there were no effects have been reported for blood pressure or inflammatory indicators (195). There are significant evidences in the scientific literature that various probiotics supplemented through various dairy products including cheese are providing health promoting properties in the consumers (176, 186). An overview of the beneficial compounds produced by microorganisms in dairy products are presented in Table 6.

**Table 6:** Beneficial and detrimental microbial compounds that can be released in fermented dairy products during fermentation and the main producer microorganisms.

Compounds	Main producer microorganisms in dairy products	References
Conjugated linoleic acid (CLA)	*Lactobacillus, Lactococcus*, and *Bifidobacterium*	(196, 197)
Oligosaccharides	*Bifidobacterium* and *Lactobacillus*	(198)
Gamma-aminobutyric acid (GABA)	*Lactococcus, Enterococcus, Lactobacillus, Pediococcus*, and *Streptococcus*	(199)
Microbial exopolysaccharides (EPS)	*Lactobacillus, Lactococcus, Pediococcus, Streptococcus thermophilus*, and *Bifidobacterium*	(200)
Vitamins (B_12_, biotin, and folic acid)	*L. plantarum, Bifidobacterium, S. thermophilus*, and *Lactobacillus delbrueckii*	(196)
Bioactive peptides		
Antioxidative	*Bifidobacterium longum* and *Lactobacillus delbrueckii*	(201)
Antimicrobial	*L. helveticus* and *L. acidophilus*	(202)
Antihypertensive	*Lactobacillus* GG*, L. helveticus*, and *S. thermophilus*	(203)
Immune modulatory	*Lactobacillus* GG	(202)

## 6 Conclusion

Fermented foods make up approximately 33% of diets in Asia, whereas they make up 60% of diets in developing countries. It has become abundantly obvious from an accumulation of studies that consuming fermented milk produces good health effects in several pathological disorders. Additionally, to improve gut health and modulate the immune system, these benefits also reduce the risk of osteoporosis, cardiovascular disease, and diabetes. Considering the growing interest showing that yogurt and fermented milk have health benefits, it may be wise to promote their frequent usage as nutritious additions to meals and tasty substitutions for other snack options. Furthermore, as fermented dairy foods, especially yogurt, kefir, cheese, and koumiss are generally accepted and consumed, they could serve as a perfect vehicle for delivering functional components, providing alternate methods for disease prevention, and promoting overall health. The stability of functional components and their interactions with the product matrix must be thoroughly investigated to ensure the functional potential of novel products throughout their commercial life. The growing body of supporting evidence from the published research is highly encouraging; it should serve as a driving force for the food industry to produce novel functional dairy products that are not currently available on the market.

## Author contributions

GS: Conceptualization, Writing – original draft. RG: Writing – review & editing. HQ: Writing – review & editing. GB: Resources, Writing – original draft. IR: Resources, Writing – review & editing. MQ: Writing – review & editing. XC: Supervision, Writing – review & editing.
